# Music interventions to reduce stress and anxiety in pregnancy: a systematic review and meta-analysis

**DOI:** 10.1186/s12888-017-1432-x

**Published:** 2017-07-27

**Authors:** Kyrsten Corbijn van Willenswaard, Fiona Lynn, Jenny McNeill, Karen McQueen, Cindy-Lee Dennis, Marci Lobel, Fiona Alderdice

**Affiliations:** 1 School of Nursing & Midwifery, Queen’s University Belfast, 97 Lisburn Road, Belfast, BT9 7BL Northern Ireland; 2 School of Nursing, Lakehead University, 955 Oliver Road, Thunder Bay, Ontario ON P7B 5E1 Canada; 3 Lawrence S. Bloomberg Faculty of Nursing, University of Toronto, 155 College St, Toronto, Ontario ON M5T 1P8 Canada; 4 Department of Psychology, Stony Brook University, Stony Brook, NY 11794 USA; 5 National Perinatal Epidemiology Unit, Nuffield Department of Population Health, University of Oxford, Oxford, OX3 7LF England

**Keywords:** Pregnancy, Psychological stress, Anxiety, Music, Systematic review, Meta-analysis

## Abstract

**Background:**

Stress and anxiety are common in pregnancy and shown to have adverse effects on maternal and infant health outcomes. The aim of this review and meta-analysis was to assess the effectiveness of music-based interventions in reducing levels of stress or anxiety among pregnant women.

**Methods:**

Six databases were searched using key terms relating to pregnancy, psychological stress, anxiety and music. Inclusion criteria were randomised controlled or quasi-experimental trials that assessed the effect of music during pregnancy and measured levels of psychological stress or anxiety as a primary or secondary outcome. Two authors independently assessed and extracted data. Quality assessment was performed using The Cochrane Collaboration risk of bias criteria. Meta-analyses were conducted to assess stress and anxiety reduction following a music-based intervention compared to a control group that received routine antenatal care.

**Results:**

Five studies with 1261 women were included. Music interventions significantly reduced levels of maternal anxiety (Standardised Mean Difference (SMD): -0.21; 95% Confidence Interval (CI) -0.39, −0.03; *p* = 0.02). There was no significant effect on general stress (SMD: -0.08; 95% CI -0.25, 0.09; *p* = 0.35) or pregnancy-specific stress (SMD: -0.02; 95% CI -0.19, 0.15; *p* = 0.80). The methodological quality of included studies was moderate to weak, all studies having a high or unclear risk of bias in allocation concealment, blinding and selective outcome reporting.

**Conclusions:**

There is evidence that music-based interventions may reduce anxiety in pregnancy; however, the methodological quality of the studies was moderate to weak. Additional research is warranted focusing on rigour of assessment, intensity of interventions delivered and methodological limitations.

**Electronic supplementary material:**

The online version of this article (doi:10.1186/s12888-017-1432-x) contains supplementary material, which is available to authorized users.

## Background

Pregnancy is a time of significant change for many women and for some this may contribute to increased stress or anxiety. The current literature uses a range of terminology to describe a perceived threat to wellbeing during the prenatal period, including the terms stress and anxiety. While both concepts are separate and can be defined individually, the terms are often used interchangeably [1, 2]. While an overall prevalence is unknown, a large US-based study found that approximately 84% of women experienced some level of stress during their pregnancy, with 6% reporting high levels [3]. A large meta-analysis of 102 studies found a prevalence of antenatal anxiety ranging from 18.2% in the first trimester to 24.6% in the third trimester, suggesting that both stress and anxiety pose concerns for a significant proportion of pregnant women [4]. A review of the literature suggests stress or anxiety in the prenatal period can include pregnancy-specific stress/anxiety, general stress/anxiety, stress related to major life events and chronic stress/anxiety.

The relationship between maternal stress and child outcomes has been of interest to researchers and health professionals for numerous years [5]. Stress during pregnancy has been linked to numerous adverse child outcomes such as poor cognitive development, autism, and schizophrenia [5]. There is also evidence to suggest that stress may have different effects depending on the trimester in which it was experienced. For example, severe stress in the first trimester has been associated with congenital malformations [5] while stress later in pregnancy may have a more negative effect on motor development [6]. This ever-growing body of research highlights the need to examine effective interventions to reduce stress during pregnancy and potentially prevent negative maternal and infant outcomes.

Music has become an increasingly popular intervention as it is low cost, easily accessible, and has high acceptability among users. Music-based interventions can vary in the amount of participant involvement and can be classified as either passive (e.g. listening to music) or active (e.g. lessons, group workshops or therapy) [7]. Several systematic reviews suggest that music-based interventions may help reduce anxiety and stress in diverse populations [8–10]. One Cochrane systematic review which included 26 trials and 1369 participants found music significantly reduced anxiety, blood pressure and heart rate among those hospitalised after a myocardial infarction [8]. Bradt et al. also found in their systematic review of 30 trials music significantly improved outcomes for cancer patients including a reduction in anxiety [9]. Lastly, a Cochrane systematic review of five randomized controlled trials conducted by Maratros et al. found music therapy may also improve mood among those diagnosed with depression [10]. Three systematic reviews on the effect of non-pharmacological interventions on maternal distress were identified [11–13] of which none specifically focused on music interventions antenatally.

Arabin and Jahn [14] conducted a study examining pregnant women’s music preferences in relation to passive listening of music to active singing and performing music. They found that of the 500 women, 72.2% listened to music daily or at least once per week, and 48.5% would be interested in taking part in some form of music programme [14]. These results suggest music may be an acceptable health promoting intervention among pregnant women.

The aim of this systematic review was to assess the effect of music-based interventions offered in addition to routine antenatal care in comparison to routine antenatal care alone/other comparison groups in reducing stress or anxiety among pregnant women.

## Methods

This review followed the Preferred Reporting Items for Systematic Reviews and Meta-Analyses (PRISMA) guidelines [15]. Six electronic databases were systematically searched for articles published from 1978 through to April 2016 and included the Cumulative Index to Nursing and Allied Health Literature (CINAHL Plus^®^ database), Cochrane Central Register of Controlled Trials (CENTRAL), Embase^®^, MEDLINE^®^, PsycInfo and Web of Science. Search terms were related to the population (pregnant women, pregnancy, prenatal care, mothers, antenatal care, antenatal, prenatal, maternal), type of intervention (music), outcome of interest (stress, anxiety, mental health, maternal welfare, life change events, worry, wellbeing, distress) and study design (RCTs, quasi-experimental). Details of the MEDLINE search strategy can be found in Additional file 1. In addition to database searching, relevant key journals were searched and any potentially relevant studies were cross-referenced with the records from the electronic database search to identify any additional studies for inclusion. The reference lists of papers that had met the inclusion/exclusion criteria were also searched to further identify potentially relevant studies and were cross-referenced with the results of the electronic database searches.

Studies were included if they: (1) used a randomised controlled trial or quasi-experimental design, (2) recruited women during pregnancy, (3) evaluated the use of a music-based intervention (active or passive), (4) measured antenatal psychological stress, or anxiety as the primary or secondary outcome, and (5) presented original data.

Titles and abstracts were screened for relevance to the review question. Full-texts of potential articles were then assessed against the eligibility criteria. One author (KCvW) screened all the records for eligibility, while a second author (FL) independently screened records to ensure methodological rigour. Any queries about study eligibility were discussed with two other co-authors to reach consensus (JMcN, FA).

A data extraction form was developed by the authors to record relevant data from the included studies and based on the recommended tool by the Cochrane Collaboration [16]. The form was piloted with several studies and minor adjustments were made to ensure all relevant data were extracted. Two authors extracted and entered data for analysis (KCvW, FL).

The Cochrane Collaboration tool for assessing risk of bias was used to assess the quality of the included studies [16]. This assessment tool considers the internal validity of studies by examining the sequence generation, blinding of participants, personnel and outcome assessors, and whether there was selective outcome reporting. Following the Cochrane Collaboration’s guidelines, studies were given one of four ratings; a low risk of bias in which all criteria were thought to have a low risk of bias, a moderate risk of bias in which several of the risk of bias criteria had been met, a high risk of bias in which the majority of the criteria were thought to have a high risk of bias. Finally, an unclear risk of bias was appointed to studies in which one or more of the criteria could not be given a high or low risk of bias due to insufficient information [16].

The main outcomes examined in the meta-analyses were (1) maternal stress, which included both general and pregnancy-specific, and (2) maternal anxiety measured post intervention. Pooled estimates using standardised mean differences (SMDs) were calculated with 95% Confidence Intervals (CIs) and analysed using a random effects model with generic inverse variance methods. If a study contained multiple intervention groups, results were combined across all eligible music intervention groups and compared with all eligible control groups. Statistical heterogeneity of the data was assessed using I^2^. A subgroup analysis was planned in relation to the medical risk status of the participants. A second subgroup analysis was planned in relation to the type of intervention (e.g. listening to music vs. making music). Sensitivity analyses were planned to assess whether results were sensitive to restricting the meta-analysis to those studies deemed at low risk of bias, as categorised by the Cochrane tool. Review Manager 5.3^®^ was used to perform the meta-analyses.

## Results

**Fig. 1:**
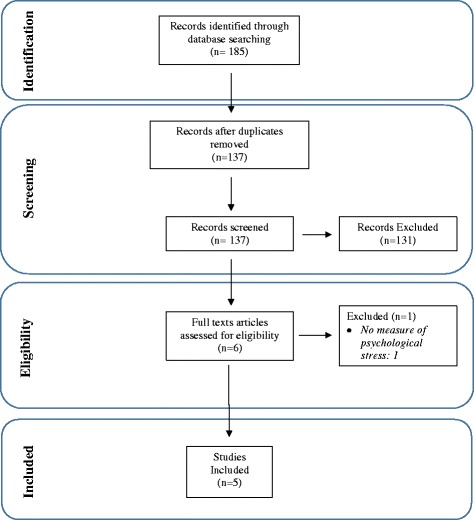
PRISMA flow chart

Database searches identified 185 records, of which 48 were duplicates (Fig. 1). Title and abstract screening identified six studies for full text assessment for eligibility. Most common reasons for study exclusion were: interventions were not music-based, intervention delivery was not during the antenatal period, and the study did not assess maternal stress, or anxiety. During the full-text assessment for eligibility, one study was excluded, as it did not measure psychological stress or anxiety.

Five studies were deemed eligible for inclusion [2, 17–20], presented in Table 1. Of the included studies, four were RCTs and one reported a quasi-experimental design with the use of a non-equivalent control group. All were published between 2008 and 2015. Four of the five studies were conducted in Asia (two in Taiwan, one in South Korea, and one in China), the fifth in the United States [17]. In total, there were 1261 participants, 602 in the intervention groups and 659 in the control groups. Study sample sizes varied, ranging from 80 participants [17] to 296 [18]. The mean age of participants ranged from 30.3 [2] to 31.4 years [17]. All five studies included women who were both primiparous and multiparous. Timing of recruitment varied from first trimester [19] to either second or third trimester [2, 17, 18], or third trimester alone [20]. Two of the studies included women who were categorised as medically high risk, defined as being hospitalised due to an obstetric diagnosis including premature rupture of membranes, preeclampsia, preterm labour, and placenta previa haemorrhage [17, 20].

**Table 1:** Summary of included studies

Study	Method	Sample	Intervention	Outcome measures and timing of assessment	Findings
Bauer et al. (2010) [17]	RCT	80 women, mean age 31.4 yrs., 24–36 weeks gestation at enrolment, medically high risk	*Intervention* *(n = 19):* 1 × 1 h with music therapist – 90% participants chose music-focused relaxation. *Intervention* *(n = 19):* 1 × 1 h relaxation intervention – 90% participants chose creative arts. *Control* *(n = 42):* waitlist attention group.	• Distress (Antepartum Bedrest Emotional Impact Inventory) *Baseline + immediately post intervention + 48–72 h after intervention*	Distress was significantly reduced by music and relaxation when compared to the control group.
Chang et al. (2008) [2]	RCT	236 women, mean age 30.3 yrs., 18-22 weeks or 30-34 weeks gestation at enrolment, medically low risk	*Intervention* *(n = 116):* routine antenatal care plus listening to music for 2 weeks for 30 min/day. 4 types of music to choose from: lullabies, classical music, nature sounds, and crystal music. *Control* *(n = 120)* = routine antenatal care.	• Stress (Perceived Stress Scale) • Anxiety (State-Trait Anxiety Inventory) *Baseline + immediately post intervention*	Music significantly reduced both stress and anxiety. However, stress was also significantly reduced in the control group.
Chang et al. (2015) [18]	RCT	296 women, aged between 24 and 41 yrs., gestational age ≥ 17 weeks at enrolment, medically low risk	*Intervention* *(n = 145):* routine antenatal care plus listening to music for 2 weeks for 30 min/day. 5 types of music to choose from: crystal music, nature sounds, classical music, lullabies and symphonic music. *Control* *(n = 151):* routine antenatal care	• Stress (Perceived Stress Scale) • Pregnancy Specific Stress (Pregnancy Stress Rating Scale) *Baseline + immediately post intervention*	Music listening did not significantly reduce stress scores; while pregnancy specific stress was significantly reduced by music.
Shin & Kim (2011) [19]	Quasi-experimental: non-equivalent control group non-synchronised design	233 women, modal age 30-34 yrs., 1st trimester at enrolment, medically low risk	*Intervention* *(n = 117):* listening to music for a single 30 min session during Transvaginal Ultrasound. Music chosen by researchers; ‘Prenatal music album with the sound of nature’ *Control* *(n = 116)*: Transvaginal Ultrasound without music.	• Anxiety (State-Trait Anxiety Inventory) • Pregnancy Specific Stress (Pregnancy Stress Scale) *Baseline + immediately post intervention*	Music significantly reduced anxiety compared to the control group. However, it did not significantly reduce pregnancy specific stress scores.
Yang et al. (2009) [20]	RCT	120 participants, “most (96.7%) were under 35 years old”, gestational age: 28-36 weeks at enrolment, medically high risk	*Intervention* *(n = 60):* usual care plus listening to music for 3 days for 30 min/day. 3 types of music to choose from: classical music, pleasant music, and Chinese folk music. *Control* *(n = 60):* usual care	• Anxiety (State-Trait Anxiety Inventory) *Baseline + immediately post Intervention*	Significantly larger reduction of anxiety in the music group than the control group.

To measure maternal stress, the Perceived Stress Scale (PSS) [21] was used by three studies [2, 18, 20] to measure general stress. The Pregnancy Stress Rating Scale (PSRS) was used by Chang et al. [18], while the Pregnancy Stress Scale was used by Shin and Kim [19]. To measure maternal anxiety, the State Trait Anxiety Inventory (STAI) [22] was used by three trials [2, 19, 20]. Bauer et al. [17] used the Antepartum Bedrest Emotional Impact Inventory (ABEII), an unpublished tool developed by the authors based on the Antepartum Hospital Stressors Inventory [23], Hospital Anxiety Depression Scale [24], Perceived Stress Scale [21], the State-Trait Anxiety Inventory [22] and the Edinburgh Postnatal Depression Scale [25]. All trials provided immediately post-intervention data and one study included a second follow-up assessment at 48–72 h post-intervention [17].

In the included studies, the outcome measures were administered pre and post intervention. In three of the studies, all outcomes measures were completed within a hospital setting [17, 19, 20] while in two studies the pre-intervention questionnaire was administered in a hospital setting and post-intervention questionnaire was sent by mail [2, 18].

Table 2 presents a summary table of the risk of bias. Overall, the trials were not well described. Within the domain of sequence generation two of the five studies were deemed to have a high risk of bias [18, 19], the remaining three had a low risk of bias [2, 17, 20]. Under allocation concealment, one study was deemed at high risk of bias [19], the other four studies had an unclear risk of bias [2, 17, 18, 20]. Within the domain of blinding participants, personnel and outcome assessors two studies had a high risk of bias [17, 19], while three had an unclear risk of bias. For the domain of incomplete outcome data all studies were deemed to be at low risk of bias [2, 17–20]. Selective outcome reporting was deemed to be unclear in all five studies [2, 17–20].

**Table 2:**
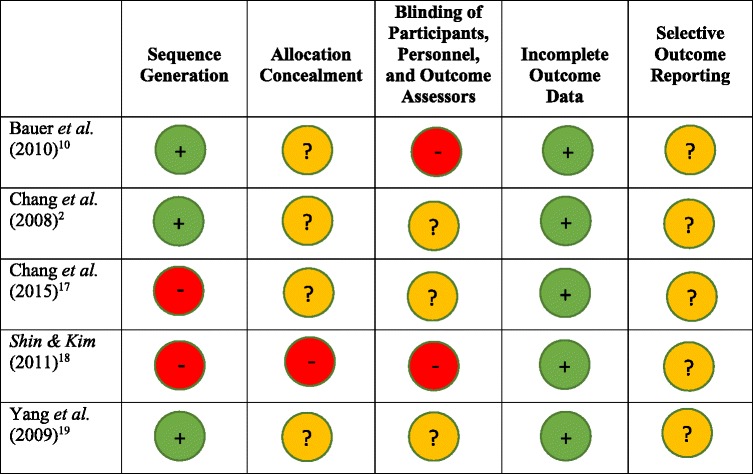
Summary of risk of bias

While all of the studies compared the intervention group to a control group, one study incorporated a three arm design, with an additional group receiving a recreational-based intervention [17]. Four trials had usual care for the control [2, 18–20], while one trial used a wait-list control group.

All the interventions involved listening to music. Shin and Kim [19] required women to listen to a ‘prenatal music album with the sound of nature’ in the examination room before, during and after a transvaginal ultrasound (TVUS). Chang et al. [2, 18] gave women the option of lullabies, classical music, nature sounds, or crystal music, all of which mimicked the human heart rate. Similarly, Yang et al. [20] chose “slow rhythm, low or moderate pitch, and a harmonious melody” music with a choice of Chinese folk, pleasant (contemporary) or classical. These four studies reported only listening to music, and did not combine music with other types of stress reduction interventions [2, 18–20]. In the Bauer et al. [17] trial, women chose to listen to improvised, live, non-vocal music and received information about muscle relaxation and breathing techniques from a music therapist or recreation therapist. In relation to the acoustic features of the music used, little was described. Three studies reported that the music selected mimicked an average human heart rate (60–80 beats/min) [2, 18, 20], and Shin and Kim reported adjusting the volume to the preference of the participant [19].

Interventions varied in terms of duration and intensity across the studies. Shin and Kim [19] used a single 30 min session, Bauer et al. [17] had women participate in a 1 h session and in the Yang et al. [20] trial, women took part in three sessions of music listening, each lasting 30 min across three consecutive days. Conversely, Chang et al. [2, 18] encouraged women to listen to music for at least 30 min a day for 2 weeks.

**Fig. 2:**
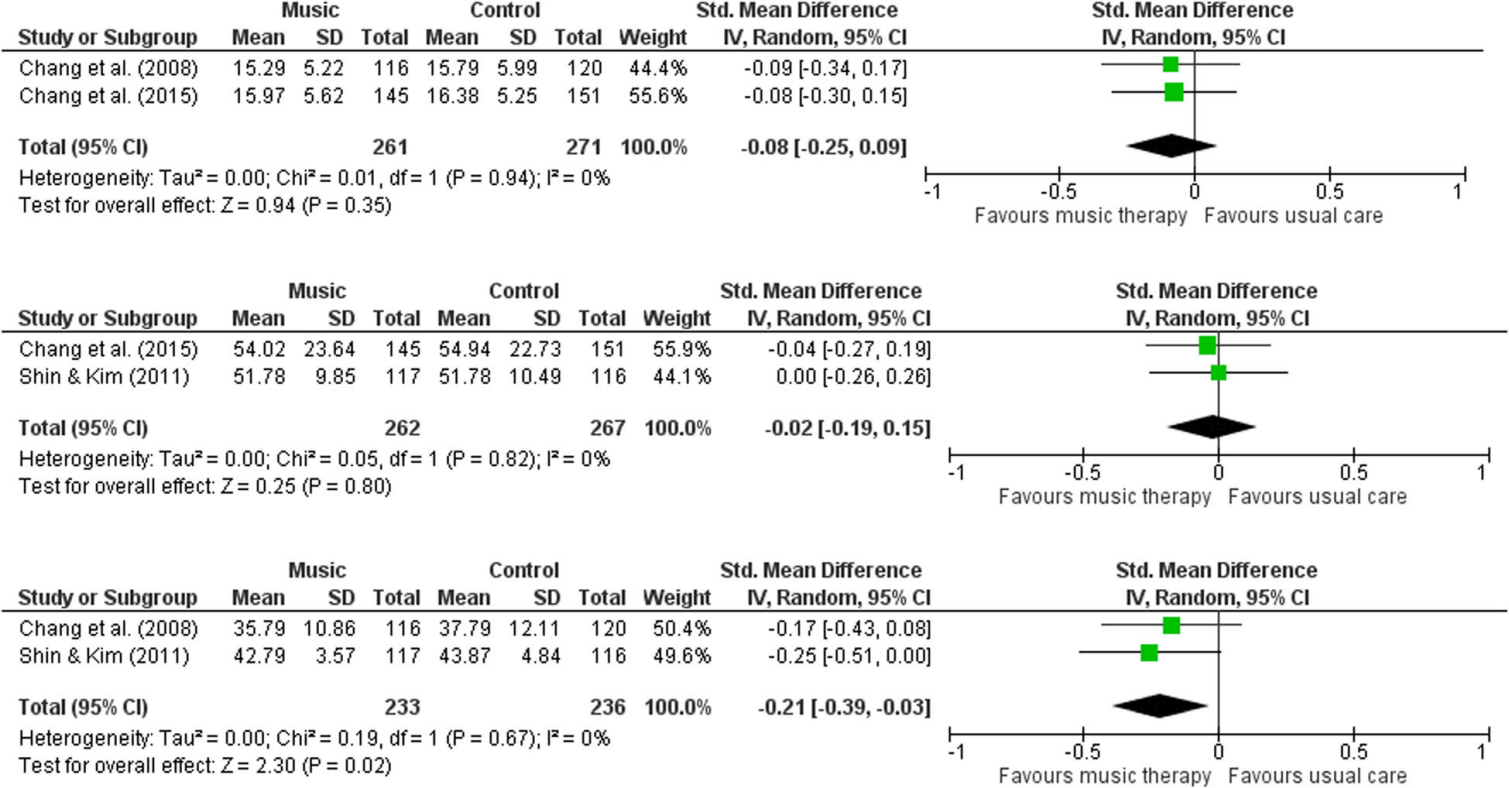
**a.** Comparison of music versus control on general stress. **b.** Comparison of music versus control on pregnancy-specific stress. **c.** Comparison of music versus control on maternal anxiety

Three studies provided data for inclusion in the meta-analysis that assessed the effect of music versus control [2, 18, 19]; two provided data on anxiety measures [2, 19], two on general stress measures [2, 18] and two on pregnancy-specific stress [18, 19]. Data from Yang et al. [20] and Bauer et al. [17] could not be included in the meta-analysis. Yang et al. [20] was excluded due to useable data not being available. Bauer et al. [17] was excluded as the measure of maternal stress was not standardised and conceptually did not fall into the review outcome categories of stress, anxiety or pregnancy-specific stress. When pooling data from the two studies in relation to levels of stress, there was no statistically significant difference between groups (SMD -0.06; 95% CI -0.20 to 0.09; *p* = 0.44) (Fig. 2a). Analysing the data from the two studies that used pregnancy-specific stress measurement tools, the meta-analysis indicated that there was no significant improvement for those in the intervention group compared to those in the control group (SMD-0.02; 95% CI -0.19 to 0.15; *p* = 0.80) (Fig. 2b). Combining the results from two studies in relation to levels of anxiety, the meta-analysis indicated those in the intervention group had a significant decrease in maternal anxiety when compared to women in the control group (SMD -0.21; 95% CI -0.39 to −0.03; *p* = 0.02) (Fig. 2c). The two planned subgroup analyses were not feasible. The planned sensitivity analysis was not conducted as it was deemed inappropriate due to the high/unclear risk assessment of the risk of bias domains across all three studies.

## Discussion

### Principal findings

Five studies incorporating 1261 women were included in the review of which three contributed data to the meta-analysis [2, 18, 19]. Overall, music-based interventions did not show significant reduction in general maternal stress or pregnancy-specific stress. However, there is preliminary evidence to suggest music-based interventions may decrease levels of maternal anxiety immediately post-intervention. It is noteworthy that while data from the studies conducted by Yang et al. [20] and Bauer et al. [17] could not be included in the meta-analysis, results from both studies are consistent with our findings.

### Interpretation

Findings from this review are similar to those found in other healthcare populations that examined music-based interventions. The studies included used pre-recorded music and involved pregnant women listening to music; no studies that examined active music therapy were found during our detailed search. Other systematic reviews that examined music-based interventions to improve clinical outcomes included a variety of different interventions ranging from listening to pre-recorded music to having one-to-one or group sessions with a music therapist [8–10, 26, 27]. In all of these reviews [8–10, 26–28] the music-based interventions positively influenced participant outcomes.

Currently, many trials do not provide a rationale for the intervention duration or intensity. Within our review, the number of sessions ranged from a single session to 14 sessions, while the length of sessions ranged from 30 min to 1 h. This lack of consistency is also found in other reviews, with cancer patients receiving anywhere from 1 to 40 sessions and in one trial participants took part in one 150 min session [9]. The short duration and intensity of the included interventions may have limited benefits on the well being for both women and their fetuses, and longer term interventions spanning the entire antenatal period should be explored.

There is currently no strong evidence supporting the hypothesis that listening to music at varying gestational ages will have differing effects on maternal stress levels. However, there has been evidence to show that levels of stress vary during pregnancy dependent on gestation, with self-reported questionnaire scores relating to poor mood being higher in the first and third trimester than in the second trimester [29]. Therefore, there may be a possible changing effect of music dependent on gestational age.

Guidelines for music-based interventions highlight the importance of clear reporting to ensure studies can be replicated in both future research and in clinical settings [30]. These guidelines recommend reporting of the theoretical underpinnings to the intervention, the setting in which the intervention is delivered, and the intervention content (including the type of music and the person selecting the music choice). The choice of music is an important component, with stress reduction being dependent on the music preference of the participant [31]. Four of the studies included in this review refer to the choice of music having a possible influence on the effectiveness of the intervention [2, 18–20], which has also been reported within reviews of music-based interventions in other patient populations [8, 26].

Due to the multidimensional nature of maternal stress, this outcome is often underestimated if the measure does not take into account the unique aspects of maternal stress or anxiety [32] and the psychological and social challenges experienced during pregnancy [33]. In recent decades, a number of standardised, reliable and valid antenatal measures have been developed that can be included in future research, for example, the Prenatal Distress Questionnaire (PDQ) [34] and the Pregnancy-related Anxiety Questionnaire (PRAQ) [35]. A review by Alderdice, Lynn and Lobel [36] showed both the PDQ and the PRAQ to have both internal reliability, with Cronbach’s alpha scores ranging from 0.80–0.81 and 0.95, respectively, and convergent validity, with significant correlations with general stress or anxiety measurement tools.

### Strength and weaknesses

Various outcome measures were used, thus limiting our ability to determine clinical significance. While four of the included studies used routine antenatal care for the control group [2, 18–20], no trial described what constituted routine antenatal care. Given that these studies were conducted in Taiwan, China, and South Korea, significant variations in antenatal care in comparison to the UK, USA or Europe for example would be expected.

Although this review demonstrates that music may reduce maternal anxiety levels antenatally, the poor quality of the studies included in this review suggests the results should be interpreted with caution. All five studies in the review were given an unclear risk of bias due to a lack of published trial details. In addition to this, the meta-analysis should be interpreted with caution. This is due to the small number of studies included in the meta-analysis, the variation between these studies in terms of participants’ characteristics, the design of the interventions, and the outcome measurements used.

### Research implications

Within this current review the duration and frequency of sessions vary greatly, with some interventions ranging from a single session to multiple sessions. However, there is currently no information regarding the optimal duration for a session, the number of sessions to deliver or the timeframe needed, particularly in terms of gestational period, to reduce stress levels in pregnant women. Ascertaining the optimal dosage for a music-based intervention is a recommended area for further research.

A common finding among previous systematic reviews that examined music-based interventions was the poor methodological quality of the majority of included trials. The characteristics of the included studies and limitations in the trials’ designs emphasise the need for further research in this area. Future research should consider the preferences of participants in the type of music they listen to [2, 18–20], the duration and frequency of the intervention being delivered [17–19], assess outcomes over a longer follow up [2, 17, 20] and also include physiological measures of distress as an outcome of interest [17, 19].

### Clinical implications

Better Births, a UK report published as part of the ‘Five Year Forward Plan’, acknowledged the significant impact that poor mental health can have on the health of both women and their children as well as the current underfunding of both perinatal and postnatal mental health care [37]. Stress and anxiety are linked with other mental health problems. For example, there is a high comorbidity between depression and anxiety, with 9.5% of women who reported having anxiety also reported having depression during their pregnancy [38]. In addition to this, women who have anxiety antenatally are more likely to have long term mental health problems, as they are at a higher risk of experiencing anxiety in the postnatal period than those who did not have antenatal anxiety [39]. Early identification and treatment of maternal anxiety and stress may lead to fewer babies being exposed and thus reducing a child’s risk for adverse outcomes.

## Conclusion

Five studies were included in this systematic review of music-based interventions to reduce stress and anxiety in pregnant women. Music interventions did not significantly reduce general or pregnancy-specific stress; however, there is preliminary evidence to suggest they may reduce anxiety during pregnancy. Methodologically strong randomized controlled trials with clearly articulated interventions, agreed core outcomes and standardised reporting measures are needed.

## Additional file

Additional file 1: MEDLINE search strategy. Description of data: Full details of MEDLINE search strategy. (DOCX 17 kb)
