# Supplementary material for: Music interventions to reduce stress and anxiety in pregnancy: a systematic review and meta-analysis
Source: BMC Psychiatry. 2017 Jul 27;17:271. doi: 10.1186/s12888-017-1432-x (PMC5531014; doi:10.1186/s12888-017-1432-x)
Supplement: Additional file 1: — MEDLINE search strategy. Description of data: Full details of MEDLINE search strategy. (DOCX 17 kb) [file 12888_2017_1432_MOESM1_ESM.docx]

# **MEDLINE Search Strategy**

1. Pregnancy/
2. Pregnant women/
3. Prenatal Care/
4. Mothers/
5. Antenatal Care.mp.
6. Antenatal.mp.
7. Prenatal.mp.
8. Maternal.mp.
9. 1 or 2 or 3 or 4 or 5 or 6 or 7 or 8
10. Stress, Psychological/
11. Anxiety/
12. Mental Health/
13. Maternal Welfare/
14. Life Change Events/
15. Worry.mp.
16. Worries.mp.
17. Wellbeing.mp.
18. Well-being.mp.
19. Wellness
20. Distress.mp.
21. Pregnancy Complications/px [Psychology]
22. 10 or 11 or 12 or 13 or 14 or 15 or 16 or 17 or 18 or 19 or 20 or 21
23. Intervention Studies/
24. Random Allocation/
25. Randomized Controlled Trial/
26. Primary Intervention.mp.
27. Controlled Clinical Trial/
28. RCT.mp.
29. 23 or 24 or 25 or 26 or 27 or 28
30. 9 and 22 and 29
31. Limit 30 to (English language and humans and yr=”1978-Current”)
32. 31 and music.mp.
